# How to Mesmerize

**Published:** 1882-11

**Authors:** 


### HOW TO MESMERIZE.

A recent writer on the mysteries of mesmerism says: "I lay it down as a matter which can be verified by all who are curious enough to try it, that the mesmeric conditions can be produced without the supposition of a subtle fluid, without the use of the cabalistic passes of the mesmerist, without the bouquet, the magnetic rod, or any of the mysterious means employed by the professionals to heighten the effect of what would be too simple and too unattractive if performed straightforwardly. The directions are these: Place the person to be operated on naturally in a chair. With your left hand suspend by a string, about a foot from the eyes, some small object, a dark marble, or a bright steel ball, or a diamond—it matters not what, though something bright is, perhaps, preferable. Direct the subject to fasten his eyes and concentrate his attention on the object. Slowly raise your left hand until the object is as far above the eyes of the patient as is compatible with his gazing steadily at it. Watch his eyes. At first you will see the pupils contract, but after a few seconds they will expand rapidly. When they are at the point of greatest expansion, move the first two fingers of your right hand from the object directly toward the eyes, the fingers being separated, fork-like, to embrace both eyes. As the fingers approach the eyes will close, and the subject will be unable to open them. After a quarter of a minute the subject will be thoroughly under control, so that the operator may make him believe whatever he tells him. Left quiet, the subject will sink into a profound torpor, during which his ears may be pierced, his cheeks sewed to his nose, and even a finger cut off without pain. To arouse him—and this is an important step—wind, either from a hand-bellows or a fan, should be directed against his eyes, or else his eyes should be tickled with a feather. The rationale of the method is simple. The fixed stare of the subject fatigues his retinal nerves, and, when the operator's fingers approach the eyelids close, as eyelids always do when the eyes are threatened. But the fatigue of the nerves has produced muscular fatigue as well, transient paralysis in the eyelids has resulted, and they cannot be opened. The eyes being then closed, the delicate frontal nerves being exhausted, and the mind made vacant by monotonous attention to one object, the patient is in a condition to fall asleep—and he does fall asleep. He is now ready to dream. The only thing remaining to do is to make him dream. But how is this to be effected? Dreaming, as has long been determined, is the result of external suggestion. Dr. Gregory, to illustrate, having been thinking of Vesuvius, went to bed with a jug of hot water at his feet and dreamed that he was climbing the sides of the burning mountain. Dr. Reid read a book on the Indians, put a blister to his head on retiring, and thought in his sleep that he was being scalped. Both of the dreams, as all others are, were caused by suggestions offered externally. These suggestions, being received while the directing power—the common sense of the mind—was in abeyance owing to sleep, were interpreted very erroneously, yet according to plain laws of association. The hot water in the one case called up the previous subject of thought —Vesuvius; the stinging blister, in the other, the equally stinging scalping-knife. It is now easy to see how the sleeping subject may be made to accept as truth whatever he is told."

---

NEVER answer questions in general company that have been put to others.

THE reaction against materialism in science and dogma in religion has set in. Science must become imbued with the spirit of religion, and religion must adopt the methods of science.—*Jewish Tribune.*

